# Strategies for enhancing medical student resilience: student and faculty member perspectives

**DOI:** 10.5116/ijme.5a46.1ccc

**Published:** 2018-01-12

**Authors:** Julia Farquhar, Robert Kamei, Arpana Vidyarthi

**Affiliations:** 1 Duke University School of Medicine, USA; 2 Duke-NUS Medical School, Singapore

**Keywords:** Resilience, medical students, wellness, well-being, qualitative research

## Abstract

**Objectives:**

To improve programs aimed to enhance medical student resiliency, we examined both medical student and faculty advisor perspectives on resiliency-building in an Asian medical school.

**Methods:**

In two separate focus groups, a convenience sample of 8 MD-PhD students and 8 faculty advisors were asked to identify strategies for enhancing resilience. Using thematic analysis, two researchers independently examined discussion transcripts and field notes and determined themes through a consensus process. They then compared the themes to discern similarities and differences between these groups.

**Results:**

Themes from the student suggestions for increasing resilience included “Perspective changes with time and experience”, “Defining effective advisors,” and “Individual paths to resiliency”. Faculty-identified themes were “Structured activities to change student perspectives,” “Structured teaching of coping strategies”, and “Institution-wide social support”. Students described themselves as individuals building their own resilience path and preferred advisors who were not also evaluators. Faculty, however, suggested systematic, structural ways to increase resilience.

**Conclusions:**

Students and advisors identified some common, and many distinct strategies for enhancing medical student resilience. Student/advisor discrepancies may exemplify a cultural shift in Singapore’s medical education climate, where students value increased individualism and autonomy in their education. As medical schools create interventions to enhance resilience and combat potential student burnout, they should consider individually-tailored as well as system-wide programs to best meet the needs of their students and faculty.

## Introduction

The demands of practicing medicine are significant, and high levels of stress and related burnout are widespread among medical students, residents, and physicians.^1^ Many stressors are essential to the healthcare profession and begin in medical school, continuing and increasing through training and practice.^2^^,^^3^ Stress has multiple effects on physicians and has been linked to empathy decline, decreased professionalism, fatigue, poor health, drug use, psychological distress, and increased suicide rates.^4^^,^^5^ Thus, much emphasis has been placed on increasing resiliency, previously defined in the medical education literature as " the capacity to resist or manage adversity without developing physical or psychological disabilities," during early medical school training.^6^ Resiliency has been proposed as a mediator to the stressors of medicine and may have positive long-term and far-reaching effects.^7 ^Although resilience-enhancing strategies have been proposed^8^ and some implemented, many are theory-based^9^^-^^12^ or not specific to medical school.^13^^-^^15^ Resiliency interventions include support from peers and advisors,^16^^,^^17^ cognitive behavioral techniques,^18^ student wellness programs,^19^^,^^20^ teaching specific coping and stress-management behaviors such as mindfulness,^10^^,^^21^^-^^23^ and implementing course changes that promote student collaboration rather than competition, such as pass/fail grading.^10^ Results of these interventions have generally been positive. 

Although this literature is informative, the majority of this research is based in Western settings,^7^^,^^17^^,^^22^^,^^24^^-^^26^ which may not be generalizable to other regions. Asian medical students, for example, may approach learning differently than their peers.^27^ Additionally, their stressors differ from their western counterparts as they experience higher test anxiety, and lower perceived social support.^28^ Thus, students in Asia may require a different approach to resiliency-building. The existing research into resiliency in Asia has been focused in China, and the perspective of Southeast Asian students is less well studied.^29^^-^^31^ Additionally, although the perspective of students is valuable, the suggestions of medical school faculty advisors, who advise medical students over long periods of time and can see developing trends in the medical student experience, could further inform our current understanding of medical student resilience. A better understanding of the perceptions of both medical students and their advisors about stress and resiliency in Asia may allow educators to create an effective and specific approach to resiliency-building.

Another gap in the literature is the qualitative perspective of the medical students and their advisors regarding resiliency building. Although limited open-ended surveys may suggest that advising and wellness courses may improve resilience,^32^^-^^34^ the specifics of the student experience remain unknown.

The objective of this study was to examine both medical student and advisor perspectives on resiliency-building in Asia, to determine similarities and differences in their perspectives, and thus inform resiliency interventions at the medical school level. To do so, we conducted focus groups of these two groups at Duke-NUS Medical School in Singapore and asked them to discuss strategies for enhancing medical student resilience.

## Methods

### Participants

Duke-NUS students follow a four-year graduate entry curriculum. Year 1 is a pre-clinical year during which students study basic sciences, while years 2, 3 and 4 are clinical years, spent in clinical settings and conducting research.  Students in the MD-PhD track at Duke-NUS join their classmates for years 1 and 2 and begin their PhD work in year 3, and complete their final year of clinical training after finishing their PhD program. 

The Duke-NUS “college masters” are a group of seasoned advisors selected from our faculty through a competitive process and include clinicians and physician-scientists. These individuals work within the framework of an advising system during all four years of medical training. They are trained to act as confidential counselors and advisors. Their role requires them to meet regularly with students both individually and in groups, and write recommendation letters for each of the students for residency.

This research was reviewed and approved by the National University of Singapore Institutional Review Board and determined to be exempt from full review as risk to participants was minimal, and data was analyzed anonymously. Recruitment of participants was compliant with ethical regulations of the NUS school governing body.

### Data collection

We invited MD-PhD students by email to participate in a confidential focus group exploring medical student stress and resilience. A researcher unknown to the students facilitated the focus group, first describing the purpose of the focus group then leading the students through a discussion of stress and coping, finally asking the group the question, “How do you think we could build student resilience?”  The students discussed their suggestions, and the session ended when students agreed there was no further information to share. Eight MD-PhD students from years 1 and 3 of training attended the focus group; two were female, six were male, and three were in their PhD research period.

In a separate process, we invited the “college masters” by email to participate in a focus group exploring medical student stress and resilience during an advisor training retreat. A member of the faculty known to a few of the advisors facilitated the focus group.  The facilitator first described the purpose of the focus group, and then participants were asked to individually reflect on and write down stressors in the medical school, and strategies to enhance medical student resilience, which were then discussed by the group until the advisors had no further thoughts. Eight advisors attended the advisor focus group, two women, and six men. Participants were physicians from specialties that included hematology-oncology, pathology, anesthesia, radiology, dermatology, and family medicine. All participants had at least seven years of advising experience.

At the completion of the sessions, resilience strategies from the student focus group were transcribed from recordings and entered into Microsoft Excel. Strategies from the advisory group were collected in their written form and transcribed into Microsoft Excel. Students filled out a brief demographics sheet, and information on faculty advisor participants was obtained through a review of publically available information including Curricular Vitae if applicable.

### Analysis

The focus group facilitator and co-author conducted a thematic analysis. The two data sets (student group and faculty advisor group) were analyzed separately. The authors independently read the comments from students and faculty advisors, taking notes on recurrent themes, and then jointly developed a thematic framework. Next, they independently sorted each item into themes and subthemes, after which they met to discuss and re-classify individual data points until they reached consensus. 

As a final analysis, the themes of the student set were compared to those of the advisor set by two authors. Both authors independently compared direct quotes from students and faculty advisors, as well as the themes from the earlier analysis. Similarities and differences between students’ and faculty advisors’ resilience-building suggestions were then compared using thematic analysis techniques. 

## Results

Students discussed fourteen independent topics related to enhancing resilience, many of which were thematically interrelated.  Faculty advisors identified twenty-eight strategies for enhancing student resilience. We identified three common themes to the resiliency strategies identified by students, and three common themes to the strategies identified by faculty. There were also two ways in which student and faculty suggestions differed.  Themes are discussed in detail below.

### Student strategies for increasing resilience

#### Perspective changes with time and experience

Students discussed that resilience often increases due to a change in perspective that occurs over time, and with experience. Experiences, particularly coming into contact with patients, increased their resilience by changing their opinions of what matters most in medical school. Their exam-related stress changed into an interest in becoming a good physician, and they learned to accept “failure” as part of the process medical training. They also discussed that previous life experiences, such as participating in the military, had increased the resilience of their classmates. 

“There comes a day during our careers, I think, for most of us when we realize, man, this is not just about passing exams... you know, we write exams every day due to the nature of the team-based learning program, but one of those days we realize that this is about me, developing the knowledge and activities that I need to become a good doctor in the future. And since that day onwards I think we become resilient”.  (Student 1, male, year 1)

“I feel like each, and maybe the number of experiences you have in life will sort of affect how resilient you become because it just puts everything into perspective”. (Student 2, female, year 1)

#### Defining effective advisors

Students described “effective” advisors as those with whom students identify, and who have no competing interests.  Students discussed the importance of advisors in increasing their resilience and reducing stress. They particularly appreciated guidance from older peers who understood their current experience. They also preferred advisors similar to them in interest and personality, whom they felt could give more applicable advice.

Students found it difficult to speak openly and share their struggles with advisors who evaluated them in some way, including writing letters for residency applications. They avoided honest discussion with these advisors.  They also felt that some advisors had conflicting interests in interacting with students, including better evaluations of their courses, assistance with research, and even higher pay; students did not feel supported by these teachers. Having difficult conversations with such advisors added to students’ stress and decreased their resilience.

“If you don’t resonate with that person, and you don’t share that person...you feel the opposite of what they’re trying to do. Instead of feeling inspired you feel depressed by what they’re saying.” (Student 3, male, PhD)

“I don’t think the college master is enough [of an advisor]. Because the college master writes your [residency letter] …so you don’t want to do anything or say anything funny to college masters. Because you know that they are probably evaluating you somehow.” (Student 4, female, year 1)

#### Individual paths to resiliency

Students felt that resilience was an internal process built by individual experiences and that individuals had different reasons for pursuing medicine. They started school with varying resiliency levels, due to the diversity of their prior experiences. Because of these differences, they believed that each student would have different stressors in school and a different "path" to resilience.

"...whatever works or whatever opinions work for one person are totally different for other people. (Student 3, male, PhD)

When I was first in this school some of my classmates have served the [military] for two years, so they are two years older than me, they are more mature, and they are less likely to get upset and to get stressed by the stressors. (Student 5, male, year 1)

### Faculty strategies for increasing resilience

#### Structured activities to change student perspectives

Advisors reported examples of normalizing medical student stress that include: helping students expect times of stress, sharing faculty experiences with stress and resilience, and communicating that negative feelings associated with stress were normal. They suggested sharing this information through structured activities such as lectures and the pre-existing advisor meetings. Faculty advisors also suggested reminding students to manage their expectations regarding medical school, guiding students in expecting future stressors, and using positive mental imagery during times of stress. Finally, it was suggested that faculty occasionally provide perspective by reminding students of their core values and suggesting they consider the larger context of particular stressors to reduce their effect.

#### Structured teaching of coping strategies

Skill-building suggestions included developing programs and strategies to assist students in building coping skills to manage the stress of medical training. These "strategies" would target time management skills, work-life balance skills, study strategies, communication skills, managing relationships, and reflection skills.

#### Institution-wide social support

Suggestions in this theme included any mention of support and advice from others. Advisors believed that increased social support, in both individual and group settings, would improve students' resilience. Advisors recommended recruiting faculty who were open to sharing their challenges with students and suggested an "open-door policy" where students could speak individually with advisors at any time. In group settings, our advisors suggested that the school support and facilitate group relationships and community-building activities. Finally, they recommended that our administration set up a structured process for students to communicate distress.

### Comparison of student and advisor suggestions

#### Students value individual connections; advisors want system change

Both groups stressed the importance of advice and guidance in building resilience over time. However, the students focused on small-scale, peer advising and preferred to hear from people similar to them- either older students or physician advisors with similar personalities and experiences. The advisors instead suggested large-scale ways that the school administration and the advisors could support students, such as "open-door" policies and community-building activities. A notable absence from advisors was a lack of discussion about who would make an appropriate advisor.

#### Resiliency is individualized for students; advisors recommend group skill-building

Students discussed resilience as an internal construct that increases over time with perspective and experience and described it as a process specific to each individual. Faculty advisors made concrete suggestions for tools and strategies that they thought could increase resilience. For example, both groups stressed the importance of perspective change over time in building resilience. However, students discussed perspective change as inevitable with time and experience. Faculty advisors thought that certain activities, including providing guidance on common challenges and disclosing their own journeys through medicine, could cause perspective change.

## Discussion

In this pilot focus group study of students and faculty advisors at a medical school in Singapore, our students and advisors provided some of the first experience-driven suggestions for increasing students' resilience. Students and advisors similarly discussed the need for personal perspective change, social support, and mentorship. However, students and advisors differed in their suggestions for the approach to mentorship and the individuality of resilience.

Advising programs in Western countries have been successful in improving resilience.^13^^,^^16^^,^^17^ These programs focus on student-advisor relationships and generally align with the idea of "functional advising" where there is a structural, interactional, and time-based relationship between the advisor-student pair that is focused on the needs of the student, ignoring hierarchy and status.^35^ Students and advisors in Singapore similarly value the role of mentorship, as both groups in our study notably discussed its importance without prompting. However, students and advisors had very different suggestions for sources of mentorship. Students preferred unbiased advisors, with no involvement in the grading process, as well as peer advisors; advisors suggested institutional changes to advising programs. This contrasting view may reveal insights into the current state of advising in Asia, where advisors may not develop personal relationships over time with students, or put their needs before those of the student.^36^ There may be a more paternalistic, hierarchical structure to the medical system in Singapore and some other Asian countries^37^^,^^38^ than in some traditional Western cultures, which could explain why students prefer to avoid sharing their concerns with advisors who are also physician educators and evaluators.^39^^,^^40^ Combining these suggestions, an effective advising program would offer students peer advisors who do not act as evaluators and provide structures and activities to support students.

Another interesting finding of this study was the difference between student and advisor beliefs about the process of building resilience. Medical students felt that resilience was an individual process, built over time through experience and perspective change. They did not see a place for school-wide resilience-building activities. Faculty advisors, however, proposed school-wide solutions that could increase resiliency in larger groups of students. One possible explanation for this discrepancy is a generational difference between students and faculty.

The current generation of medical students in Western countries has expressed an interest in individualism and autonomy in their learning^41^^,^^42^ and have reported feeling that attending physicians/consultants, a different generation of doctors, do not share that interest.^43^ Students in Singapore may be reporting a similar desire for autonomy as they build resilience, whereas their advisors from a different generation value collective skill-building. Singapore has been considered a collectivist society, valuing the group over the individual when compared to individual-oriented societies that predominate in Western countries.^44^ However, due in part to increased globalization,^45^ the younger generation of students in Singapore may have a more individualistic approach than earlier generations, as has been described in China, another traditionally collectivist society.^46^ Further research comparing students' and advisors' attitudes towards the roles of the individual and the institution in medical training will be valuable in further explaining this difference.

A limitation of our study is different focus group design between our student and advisory groups, with written suggestions in the advisory group and spoken comments in the student group. While this limits our ability to compare findings between the two focus groups, each group individually raised important information. Additionally, in each group, we created a methodology using group dialogue that continued until no further ideas were suggested. We conducted our study with small samples at one site, which is a medical school in Singapore and as such our findings may be more specific to our medical school. However, our site, with its diversity in students, teaching methodology, and international collaboration, may have been an asset to revealing cultural challenges that will be faced by other medical schools in the future and suggests that similar analyses may be utilized by other schools to identify stressors unique to their set.

In the future, a multi-site study using random selection and a larger group of participants may be able to further characterize the views of students and their faculty advisors in medical schools in Asia. This study will be important, as our findings suggest significant differences in their perspectives. For example, educators have advocated for increased student autonomy and self-determination in their learning experience^47^^-^^50^ and could consider resiliency-building to be similarly individualistic. Programs to improve student resiliency are often designed by faculty without student input, which will limit program impact. By designing the program, at the outset, with the student voice in mind, resiliency may be improved. Given the differences in perspectives between faculty and students, further exploring the underlying causes of the variations may improve not only resiliency programs but all medical student curricula.

## Conclusions

We characterized students' and faculty advisors' collective experiences with medical student stress and resilience in an Asian population. While it was exploratory research, our study was the first to explore faculty opinions of resiliency and the first to do so in a diverse, multinational medical school in Asia. Students and their advisors share some ideas in regard to resilience building, such as the value of perspective change and mentorship, but differ on the role of the individual compared to the institution to meet this goal. Our findings emphasize diverse, and at times divergent opinions that taken together may inform new activities for increasing medical student resilience. The new findings revealed by our exploratory study are a foundation for further research in the area of medical student resilience in general, and specifically in Asia.

### Acknowledgements

We would like to thank our student and advisory participants for their role in our study. Funding for this study came from the Duke-NUS Education Innovation Grant.

### Conflict of Interest

The authors declare that they have no conflict of interest.

## References

[1] Ishak W, Nikravesh R, Lederer S, Perry R, Ogunyemi D, Bernstein C (2013). Burnout in medical students: a systematic review.. Clin Teach.

[2] Ishak WW, Lederer S, Mandili C, Nikravesh R, Seligman L, Vasa M, Ogunyemi D, Bernstein CA (2009). Burnout during residency training: a literature review.. J Grad Med Educ.

[3] Benbassat J, Baumal R, Chan S, Nirel N (2011). Sources of distress during medical training and clinical practice: Suggestions for reducing their impact.. Med Teach.

[4] Firth-Cozens J (2001). Interventions to improve physicians' well-being and patient care.. Soc Sci Med.

[5] Hojat M, Vergare MJ, Maxwell K, Brainard G, Herrine SK, Isenberg GA, Veloski J, Gonnella JS (2009). The devil is in the third year: a longitudinal study of erosion of empathy in medical school.. Acad Med.

[6] Campbell-Sills L, Cohan SL, Stein MB (2006). Relationship of resilience to personality, coping, and psychiatric symptoms in young adults.. Behav Res Ther.

[7] Dyrbye L, Shanafelt T (2012). Nurturing resiliency in medical trainees.. Med Educ.

[8] Zwack J, Schweitzer J (2013). If every fifth physician is affected by burnout, what about the other four? Resilience strategies of experienced physicians.. Acad Med.

[9] Shapiro SL, Shapiro DE, Schwartz GE (2000). Stress management in medical education: a review of the literature.. Acad Med.

[10] Dyrbye LN, Thomas MR, Shanafelt TD (2005). Medical student distress: causes, consequences, and proposed solutions.. Mayo Clin Proc.

[11] Wald HS, Anthony D, Hutchinson TA, Liben S, Smilovitch M, Donato AA (2015). Professional identity formation in medical education for humanistic, resilient physicians: pedagogic strategies for bridging theory to practice.. Acad Med.

[12] Outram S, Kelly B (2014). "You teach us to listen,... but you don't teach us about suffering": self-care and resilience strategies in medical school curricula.. Perspect Med Educ.

[13] Rogers D (2016). Which educational interventions improve healthcare professionals' resilience?. Med Teach.

[14] Aboalshamat K, Hou XY, Strodl E (2015). The impact of a self-development coaching programme on medical and dental students' psychological health and academic performance: a randomised controlled trial.. BMC Med Educ.

[15] Goldhagen BE, Kingsolver K, Stinnett SS, Rosdahl JA (2015). Stress and burnout in residents: impact of mindfulness-based resilience training.. Adv Med Educ Pract.

[16] Howe A, Smajdor A, Stöckl A (2012). Towards an understanding of resilience and its relevance to medical training.. Med Educ.

[17] Moir F, Henning M, Hassed C, Moyes SA, Elley CR (2016). A Peer-Support and Mindfulness Program to Improve the Mental Health of Medical Students.. Teach Learn Med.

[18] Sood A, Prasad K, Schroeder D, Varkey P (2011). Stress management and resilience training among Department of Medicine faculty: a pilot randomized clinical trial.. J Gen Intern Med.

[19] Drolet BC, Rodgers S (2010). A comprehensive medical student wellness program--design and implementation at Vanderbilt School of Medicine.. Acad Med.

[20] Slavin SJ, Schindler DL, Chibnall JT (2014). Medical student mental health 3.0: improving student wellness through curricular changes.. Acad Med.

[21] Gordon JS (2014). Mind-body skills groups for medical students: reducing stress, enhancing commitment, and promoting patient-centered care.. BMC Med Educ.

[22] Thomas SE, Haney MK, Pelic CM, Shaw D, Wong JG (2011). Developing a program to promote stress resilience and self-care in first-year medical students.. Can Med Educ J.

[23] Aherne D, Farrant K, Hickey L, Hickey E, McGrath L, McGrath D (2016). Mindfulness based stress reduction for medical students: optimising student satisfaction and engagement.. BMC Med Educ.

[24] Dyrbye LN, Power DV, Massie FS, Eacker A, Harper W, Thomas MR, Szydlo DW, Sloan JA, Shanafelt TD (2010). Factors associated with resilience to and recovery from burnout: a prospective, multi-institutional study of US medical students.. Med Educ.

[25] Haglund ME, aan het Rot M, Cooper NS, Nestadt PS, Muller D, Southwick SM, Charney DS (2009). Resilience in the third year of medical school: a prospective study of the associations between stressful events occurring during clinical rotations and student well-being.. Acad Med.

[26] Kjeldstadli K, Tyssen R, Finset A, Hem E, Gude T, Gronvold NT, Ekeberg O, Vaglum P (2006). Life satisfaction and resilience in medical school--a six-year longitudinal, nationwide and comparative study.. BMC Med Educ.

[27] Tavakol M, Dennick R (2010). Are Asian international medical students just rote learners?. Adv Health Sci Educ Theory Pract.

[28] Henning MA, Hawken SJ, Krägeloh C, Zhao Y, Doherty I. Asian medical students: quality of life and motivation to learn. Asia Pac Educ Rev. 2011;12(3):437-45.

[29] Shi M, Wang X, Bian Y, Wang L (2015). The mediating role of resilience in the relationship between stress and life satisfaction among Chinese medical students: a cross-sectional study.. BMC Med Educ.

[30] Zhao F, Guo Y, Suhonen R, Leino-Kilpi H (2016). Subjective well-being and its association with peer caring and resilience among nursing vs medical students: A questionnaire study.. Nurse Educ Today.

[31] Peng L, Zhang J, Li M, Li P, Zhang Y, Zuo X, Miao Y, Xu Y (2012). Negative life events and mental health of Chinese medical students: the effect of resilience, personality and social support.. Psychiatry Res.

[32] Kötter T, Pohontsch NJ, Voltmer E (2015). Stressors and starting points for health-promoting interventions in medical school from the students' perspective: a qualitative study.. Perspect Med Educ.

[33] Lee J, Graham AV (2001). Students' perception of medical school stress and their evaluation of a wellness elective.. Med Educ.

[34] Dossett ML, Kohatsu W, Nunley W, Mehta D, Davis RB, Phillips RS, et al. A medical student elective promoting humanism, communication skills, complemen-tary and alternative medicine and physician self-care: an evaluation of the HEART program. Explore (NY). 2013;9(5):292-8. 10.1016/j.explore.2013.06.003 PMC3876728 24021470

[35] Sambunjak D, Marusić A (2009). Mentoring: what's in a name?. JAMA.

[36] Ynalvez R, Garza-Gongora C, Ynalvez MA, Hara N (2014). Research experiences and mentoring practices in selected east Asian graduate programs: predictors of research productivity among doctoral students in molecular biology.. Biochem Mol Biol Educ.

[37] Claramita M, Nugraheni MD, van Dalen J, van der Vleuten C (2013). Doctor-patient communication in Southeast Asia: a different culture?. Adv Health Sci Educ Theory Pract.

[38] Claramita M, Dalen JV, Van Der Vleuten CP (2011). Doctors in a Southeast Asian country communicate sub-optimally regardless of patients' educational background.. Patient Educ Couns.

[39] Nagata-Kobayashi S, Sekimoto M, Koyama H, Yamamoto W, Goto E, Fukushima O, Ino T, Shimada T, Shimbo T, Asai A, Koizumi S, Fukui T (2006). Medical student abuse during clinical clerkships in Japan.. J Gen Intern Med.

[40] Mori SC (2000). Addressing the mental health concerns of international students. Journal of Counseling and Development.

[41] Edafe O, Brooks WS, Laskar SN, Benjamin MW, Chan P (2016). Impact of a novel teaching method based on feedback, activity, individuality and relevance on students' learning.. Int J Med Educ.

[42] Frost HD, Regehr G (2013). "I am a doctor": negotiating the discourses of standardization and diversity in professional identity construction.. Acad Med.

[43] Seabrook MA (2004). Clinical students' initial reports of the educational climate in a single medical school.. Med Educ.

[44] Lau  S (1992). Collectivism's individualism: value preference, personal control, and the desire for freedom among Chinese in Mainland China, Hong Kong, and Singapore.. Personality and Individual Differences.

[45] Harden RM (2006). International medical education and future directions: a global perspective.. Acad Med.

[46] Kolstad A, Gjesvik N (2014). Collectivism, individualism, and pragmatism in China: implications for perceptions of mental health.. Transcult Psychiatry.

[47] Orsini C, Evans P, Jerez O (2015). How to encourage intrinsic motivation in the clinical teaching environment?: a systematic review from the self-determination theory.. J Educ Eval Health Prof.

[48] Williams GC, Deci EL (1998). The importance of supporting autonomy in medical education.. Ann Intern Med.

[49] Lyness JM, Lurie SJ, Ward DS, Mooney CJ, Lambert DR (2013). Engaging students and faculty: implications of self-determination theory for teachers and leaders in academic medicine.. BMC Med Educ.

[50] Kusurkar R, Croiset G (2014). Electives support autonomy and autonomous motivation in undergraduate medical education.. Med Teach.

